# Advanced Strategies for Developing Vaccines and Diagnostic Tools for African Swine Fever

**DOI:** 10.3390/v15112169

**Published:** 2023-10-28

**Authors:** Jong-Woo Lim, Thi Thu Hang Vu, Van Phan Le, Minjoo Yeom, Daesub Song, Dae Gwin Jeong, Song-Kyu Park

**Affiliations:** 1 Department of Veterinary Medicine Virology Laboratory, College of Veterinary Medicine and Research Institute for Veterinary Science, Seoul National University, Seoul 08826, Republic of Korea; nanobiolim@snu.ac.kr (J.-W.L.); virusxnox@snu.ac.kr (M.Y.); sds@snu.ac.kr (D.S.); 2 College of Pharmacy, Korea University, Sejong 30019, Republic of Korea; moonrtd@gmail.com; 3 Department of Veterinary Microbiology and Infectious Diseases, Faculty of Veterinary Medicine, Vietnam National University of Agriculture, Hanoi 131000, Vietnam; letranphan@vnua.edu.vn; 4 Bionanotechnology Research Center, Korea Research Institute of Bioscience and Biotechnology, Daejeon 34141, Republic of Korea; 5 Bio-Analytical Science Division, University of Science and Technology, Daejeon 34113, Republic of Korea

**Keywords:** African swine fever virus, vaccine, diagnostics, genetically engineered vaccine platforms, point-of-care

## Abstract

African swine fever (ASF) is one of the most lethal infectious diseases affecting domestic pigs and wild boars of all ages. Over a span of 100 years, ASF has continued to spread over continents and adversely affects the global pig industry. To date, no vaccine or treatment has been approved. The complex genome structure and diverse variants facilitate the immune evasion of the ASF virus (ASFV). Recently, advanced technologies have been used to design various potential vaccine candidates and effective diagnostic tools. This review updates vaccine platforms that are currently being used worldwide, with a focus on genetically modified live attenuated vaccines, including an understanding of their potential efficacy and limitations of safety and stability. Furthermore, advanced ASFV detection technologies are presented that discuss and incorporate the challenges that remain to be addressed for conventional detection methods. We also highlight a nano-bio-based system that enhances sensitivity and specificity. A combination of prophylactic vaccines and point-of-care diagnostics can help effectively control the spread of ASFV.

## 1. Introduction

African swine fever (ASF) is a lethal contagious disease that affects both wild and domestic swine and adversely affects the global swine industry. Acute forms, which result in high morbidity and mortality rates, have been described and are accompanied by several clinical manifestations [1,2]. Depending on the local viral strain and host susceptibility, subacute, chronic, subclinical, or low-level form diseases have also been described. The ASF virus (ASFV) has remained endemic in Africa since its identification in Kenya in 1921, and genotype I was notably prevalent in Europe during the early 1960s [3,4]. From the mid-1990s, ASFV genotype I remained endemic in only Sardinia. In 2007, ASFV re-emerged in Georgia and was then transmitted to some eastern European countries, such as Lithuania, Poland, Russia, Ukraine, and Belarus, followed by some Western European countries, including Belgium, in 2018 [5,6]. In August 2018, the outbreak and spread of ASFV in China were reported; numerous cases of ASF were detected in wild boar and domestic pigs [7,8,9]. Acute ASFV infection can cause mass mortality, while moderate- and low-pathogenic ASFV cause a persistent disease state that is difficult to terminate in the pig population; therefore, viable strategies to contain it include wild boar surveillance, local isolation, and culling. ASF is still an ongoing problem, and new ASFV strains have emerged. Although commercial vaccines have recently been developed in Vietnam (Document 4870/BNN-TY dated 24 July 2023, from the Ministry of Agriculture and Rural Development, Vietnam), ASFV still causes enormous economic losses, and the aforementioned strategies continue to be recommended.

Vaccines are always the most effective preventive tools against diseases. However, structural unknowns in the ASFV genome pose a challenge for developing an effective vaccine against ASF. Many ASF vaccine platforms have been studied in vitro and in vivo; however, traditional vaccines do not provide effective protection [10,11]. New vaccines generated using advances in gene-editing techniques, such as subunit vaccines, DNA vaccines, and vector virus vaccines, have improved safety and efficacy compared to conventional vaccines. Attenuated vaccines generated using gene deletions are potential candidates with high efficacy against virulent strains. However, these vaccines face safety risks and must be thoroughly evaluated before they can be approved for widespread use. Owing to the different epidemiological characteristics in different areas, which are mainly based on different livestock conditions and quarantine policies, the use of live attenuated vaccines should also be considered. In parallel with vaccine development, strict quarantine and proactive monitoring policies should be maintained to prevent ASF.

To counteract and control the ASFV, early detection using laboratory diagnosis is mainly presupposed and essential. Depending on the virulence of the strain and the host immune system, clinical symptoms and pathological conditions vary widely; consequently, ASFV could be misdiagnosed as porcine hemorrhagic diseases such as classical swine fever, highly pathogenic porcine reproductive and respiratory syndrome (HP-PRRS), and swine erysipelas [12]. Therefore, ASFV-specific antigens or DNA and antibodies must be accurately detected for laboratory tests of ASFV. Several ASFV diagnostic techniques are recommended by the World Organisation for Animal Health (WOAH, formerly OIE) and applied for diverse research objectives worldwide: hemadsorption (HAD) tests, antigen detection by fluorescent antibody tests, conventional and real-time polymerase chain reaction (PCR), ELISA, indirect immunoperoxidase tests, and indirect fluorescent antibody tests [13]. Among these methods, PCR-based molecular diagnostics are the most reliable and provide higher sensitivity and specificity; however, they require expensive equipment, sophisticated sample preparation, and skilled assistants, rendering them unsuitable for on-site tests. Because infected swine shed and transmit the virus before clinical signs appear, rapid and accurate point-of-care (POC) portable diagnostics are required for effective quarantine and the prevention of spreading.

This review summarizes the vaccines and diagnostic technologies used to control ASFV infections. First, we discuss and highlight genetically engineered vaccine platforms, and the challenges and strategies involved in their development. Next, we focus on conventional laboratory ASFV diagnostic methods and advanced POC diagnostics, including PCR, isothermal amplification, CRISPR, and lateral flow assay (LFA). We elucidate the characteristics of vaccines and diagnostic methods, introduce novel technologies for enhancing their efficacy, and support the implementation of ASFV control strategies.

## 2. Vaccine Development Model for ASFV

### 2.1. Challenges of ASF Vaccine Development

ASFV is an enveloped virus with a large double-stranded DNA genome, varying in length between 170 and 190 kb, encoding 151–167 open reading frames (ORFs) [14]. Twenty-four genotypes of ASFV have been defined based on the *B646L* (*p72*) gene, which encodes the major capsid protein p72 [4,15,16,17]. Among them, twenty-two genotypes have been circulating only in sub-Saharan Africa; two genotypes have been detected outside of Africa, including genotype I, which has been detected in Europe, South America, and the Caribbean starting from the 1950s and recently in China, while genotype II has been widespread in several countries in Africa, Europe (the east to the west), Asia, and Americas [1,18,19,20,21,22,23,24]. In terms of virulence, ASFV strains are classified into high-, moderate-, and low-virulence strains, which cause peracute/acute, sub-acute, and chronic ASF [25].

**Figure 1:**
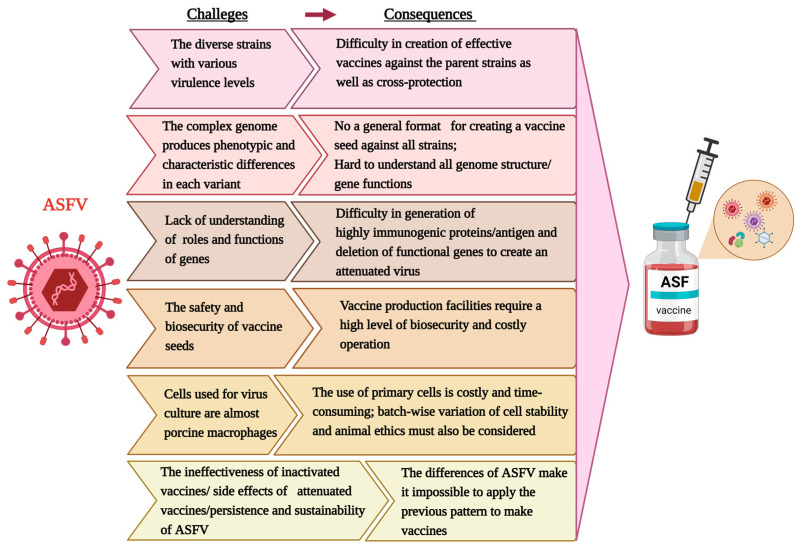
Challenges in ASF vaccine development. This figure was created with BioRender.com.

Safety and protective efficacy are the two most important criteria for vaccine approval, both of which are challenges for developing ASF vaccines (Figure 1). (i) The diversity of ASFV strains with various virulence levels makes it difficult to create effective vaccines against the parent strains as well as cross-protection. (ii) The complex genome produces phenotypic and characteristic differences in each virus strain, even within the same group. For instance, the same conserved gene is present in many strains of ASFV genotype II; deletion in one strain induces an attenuated state, but not in the other strains. Therefore, a general format for creating a vaccine seed against all strains is currently unavailable. (iii) Moreover, we still lack a full understanding of virulence-related genes, antigenic components, genes involved in attachment and infection of ASFV into host cells, and genes related to the immunosuppression, replication and assembly of viral particles. (iv) The safety and biosecurity of vaccine seeds pose further challenges. ASF seeds must be safe for vaccinated pigs, and the virus should not be transmitted or revert to virulence. Owing to the risk of ASFV in pigs, vaccine production facilities (for inactivated and live attenuated vaccines) require a high level of biosecurity and costly operation. Therefore, a standard procedure must be established to develop recombinant strains as vaccine candidates. (v) Cells used for virus culturing also pose problems in the research and production of ASF vaccines. Primary porcine cells, such as macrophages from the lung, bone marrow, blood monocytes, and kidney, have been commonly used to isolate and investigate characteristics, interactions with the host of ASFV, and produce vaccines. Porcine macrophages are the target cells of ASFV strains when they infect pigs; therefore, they are advantageous in research. However, the use of primary cells is costly and time-consuming; importantly, batch-wise variations in cell stability and animal ethics must also be considered. Several ASFV studies have been performed in cell lines such as Vero [26], COS [27], and MA104 [28,29]. Adaptations in cell lines often alter the viral genome, thereby altering the virulence and antigenic or protective capacity of the virus relative to its origin. Recently, immortalized cells from porcine macrophages, such as Immortalized porcine kidney macrophages (IPKM) [30,31], Zuckermann Macrophage-4 (ZMAC-4) [32], Plum Island porcine epithelial cells (PIPEC), and COS-1 [33,34], were reported as candidates for ASFV proliferation. The passage of ASFV-G-ΔI177L into PIPEC resulted in the deletion of a 10,842 bp gene fragment, creating a new strain, ASFV-G-ΔI177L/ΔLVR. Interestingly, ASFV-G-ΔI177L/ΔLVR retains its attenuation and protective ability against the original ASFV-G virus [35,36]. While porcine immortalized cell lines are potential candidates for developing attenuated ASF vaccines, they also require further study.

### 2.2. Traditional Vaccines

#### 2.2.1. Inactivated Vaccine

Inactivated vaccines were the first widely used vaccines against infectious diseases in animals [37]. Porcine inactivated vaccines have been commonly used to prevent diseases such as porcine parvovirus, PRRS, foot and mouth disease, porcine circovirus, etc. [38]. In principle, inactivated vaccines contain pathogens that have been killed by physical or chemical processes and adjuvants; therefore, utilizing emerging pathogens to create inactivated vaccine can offer a quick and safe response in emergencies. Autogenous vaccines produced from isolates causing outbreaks are still widely used in large-scale emergencies in the event of an endemic disease [39]. In the case of ASF, the inactivated vaccine showed a possible immune response in vaccinated animals but failed to protect pigs from the highly virulent virus. Pigs immunized with glutaraldehyde-treated ASFV showed no seroconversion and no protection against homologous virulent viruses. Challenged animals showed increased antibodies and slightly decreased levels of viremia [40,41]. To develop inactivated vaccines, different conditions such as adjuvants, inactivated conditions, and methods of virus inactivation have been developed; however, despite being considered safe, these vaccines did not provide protection against virulent ASFV strains in challenge tests [42,43,44].

#### 2.2.2. Classical Live Attenuated Vaccine

Naturally attenuated ASFV strains were isolated from chronically infected pigs. These strains do not cause clinical signs in pigs and have a certain protective ability. These are mostly type I strains, including NH/P68 [45] and OUR T88/3 [46,47,48], but some attenuated type II strains, such as Lv17/WB/Rie1, have recently been discovered as well [49]. These strains are not highly virulent but cause adverse reactions after immunization, such as slight fever, joint swelling and necrotic skin, and they have low cross-protection potential or cause chronic infection in pigs. Naturally attenuated strains have been commonly used as a model to study virulence-associated determinants, as well as their functions, to create recombinant attenuated strains.

Culture-passage is one of the most popular and effective methods for virus attenuation to produce classical attenuated vaccines in animals. The virus, after several passages in a cell line, tends to adapt to that cell line, leading to genetic changes. These mutations potentially allow the virus to proliferate better in cells and be less virulent compared to the original virus when re-infecting the host animal. Numerous vaccines have been developed using this method, yielding attenuated vaccines against diseases such as Aujeszky’s disease, porcine epidemic diarrhea (PED), and PRRS. The ASFV is also continuously passed in cell lines to produce attenuated strains. Many reports have demonstrated similar results showing that cell-adapted ASFV strains display massive genomic changes. Furthermore, multi-deletions or large DNA fragment deletions reduce the virulence of the virus while also reducing the antigenicity and ability to induce an immune response in the host [26,50]. Therefore, most of these viruses lose their protection against the parental virulent strains.

### 2.3. Genetically Engineered Vaccine Platforms

A variety of vaccine platforms have been created using novel genetic techniques, opening new opportunities to prevent infectious diseases worldwide. Subunits, live vector viruses, DNA vaccines, and recombinant live attenuated virus strains are promising candidates for ASF vaccines (Table 1).

#### 2.3.1. Subunit Vaccine

Subunit vaccines are the safest vaccines made from proteins that act as viral antigens. Virulent ASFV E75 was neutralized by p72-neutralizing mAb and sera from challenged pigs [51]. Anti-p54 and p72 antibodies inhibited 60% of ASFV attachment in Vero cells and pig macrophages, while anti-p30 antibodies inhibited more than 95% of virus internalization in both of these cells [52]. In another study, pigs were immunized with p54 and p30 and subsequently challenged with 2 × 10^5^ HAD_50_ ASFV-E75 [53]. Notably, only the group of pigs immunized simultaneously with p54 and p30 survived after the challenge, whereas pigs immunized with p54 or p30 failed to survive. In a study by Barderas et al., pigs immunized with the chimera of p54 and p30 exhibited neutralizing antibodies, survived after the challenge with a virulent ASFV, and showed a 100-fold reduction in viremia compared to the control group [54]. Neilan et al. showed that p54, p72, and p30 proteins did not demonstrate protective efficacy in animal experiments. Neutralizing antibodies delayed the onset of clinical symptoms but were not sufficient to save the pigs from challenge with 10^4^ HAD_50_ of ASFV-Pr4 [55]. This disparity can be explained by the different levels of virulence or doses of the challenge viruses. To enhance the immune response induced by ASFV antigens, Zang et al. constructed a blend of ASFV p30, p54, and p72 proteins fused with the bacterial lipoprotein OprI. This blend, formulated with the ISA206 adjuvant, robustly stimulated ASF-specific cellular and humoral immune responses in experimental pigs [56].

#### 2.3.2. DNA and Virus-Vectored (Delivery Vectors) Vaccines

DNA constructs harboring p54, p30, and extracellular domain of hemagglutinin (sHA) ASFV proteins (pCMV-UbsHAPQ) were used to immunize pigs. Twenty-five days post-challenge, 50% of the immunized pigs survived and recovered, while all pigs in the control group died eight days post-challenge [57]. Immunization with an expression library of ASFV genes in a similar pCMV-Ubiq structure (ASFV^Ublib^) resulted in 60% protection for pigs exposed to the virulent E75 strain [58]. In another study, immunization with a DNA prime containing pools of 46 or 47 antigens, coupled with a recombinant *Vaccinia* virus, led to a more pronounced decrease in the levels of ASFV viremia after the challenge compared to those observed in the control group [59]. In an alternate study, pigs were immunized with three pools of eight viral vectors carrying ASF antigens. These antigens originated from ASFV genotypes I, OUT T88/1, and Benin 1997/1. Among the vector pools, one containing *B602L*, *B646L*, *CP204L*, *E183L*, *E199L*, *EP153R*, *F317L*, and *MGF505-5R* genes achieved 100% protection for the immunized pigs following the challenge [60]. Pigs immunized with viral vectors showed strong humoral or cellular immune responses, especially with a heterologous primer boost. An adenovirus-vectored cocktail containing seven antigens with ZTS-01 adjuvant formula induced a weak antibody response; five out of nine pigs survived, and four out of five controls died after a challenge with the ASF Georgia 2007/1 strain [61]. For combined DNA and subunit vaccination, piglets were inoculated with recombinant proteins (p15, p35, p54, +/₋p17) and pcDNA constructs (with genes encoding CDv2, p72, p32, and +/₋p17 proteins), and then challenged with the virulent ASFV Arm07 strain. The results show that the vaccinated group exhibited earlier clinical signs, viremia and death, and were not protected from the virulent ASFV strain [62].

Safety is a prominent advantage of subunit vaccines, DNA vaccines, and virus-vectored vaccines. In addition, these vaccines are produced using common cell lines without a high level of biosecurity. However, to create an effective vaccine using this platform, the protective efficacy must be improved.

#### 2.3.3. Recombinant Live Attenuated Vaccine

In ASFV genomic studies, the first virulence-related genes have been reported, including *NL-S* (*DP71L*), *UK* (*DP96R*), *TK* (*K169R*), *9GL* (*B119L*), *CD2v* (*EP402R*), and *DP418R* [34,63,64,65,66,67,68]. Studies also investigated the deletion of genes related to escape from host immunity as multi-gene families (MGF). Among the five MGFs MGF-100, MGF-110, MGF-300, MGF-360, and MGF-505 in ASFV, the deletion of MGF-360 and MGF-505 created the attenuated virus and protected animals from challenge with homologous strains [69,70,71,72]. A single gene or group of genes associated with virulence was continuously determined. Simultaneously, recombinant attenuated strains were evaluated for protective efficacy in animal experiments. The deletion of a single gene or group of genes may not always create attenuated strains among different original strains. Multi-gene deletions may produce attenuated strains more efficiently than single-gene deletions.

Borca et al. contributed recombinant ASF strains related to the ASFV-Georgia strain, which was isolated in Georgia in 2007. Among those strains are ASFV-G-ΔI177L (2020), in which the conservative *I177L* gene was deleted, and ASFV-G-ΔA137R (2021) in which the *A137R* gene was deleted, to yield completely attenuated strains. Borca et al. pioneered the application of CRISPR Cas9 to create recombinant attenuated ASFV strains based on an understanding of the ASF proteome [73,74,75,76]. In June 2022, Vietnam approved the world’s first African swine fever vaccine. This recombinant attenuated ASF vaccine was developed from the ASFV-G-ΔI177L strain in porcine peripheral blood mononuclear cells (PBMC) by the National Veterinary Medicine Joint Stock Company (Navetco, Vietnam). This vaccine has been reported to be safe and efficacious in two pig breeds grown in Vietnam. ASFV-G-ΔI177L was stable and attenuated after five passages in domestic pigs [77,78]. The second ASF vaccine approved in Vietnam was AVAC ASF LIVE, which utilizes the ASFV-G-∆MGF strain. This strain was also derived from ASFV Georgia 2007/1, with the removal of six genes: *MGF505-1R*, *MGF360-12L*, *MGF360-13L*, *MGF360-14L*, *MGF505-2R*, and *MGF505-3R*. The DMAC cell line has been used to produce the AVAC ASF LIVE vaccine [79]. These attenuated vaccines are recommended for administration to healthy pigs and should not be administered concurrently with other vaccines. O’Donnell et al. published safety and protection data for homologous strains of ASFV-G-Δ9GLv (2015), ASFV-G-ΔMGF (2015), and ASFV-G-Δ9GL/UK (2017), which deleted *9GL* (*B119L*), MGFs, or combined *9GL* and *UK* genes, respectively [69,80,81]. Interestingly, the ASFV-G-Δ9GL/ΔMGF strain, which had deleted *9GL* and MGF, showed attenuated but non-protective effects compared to the parental strain [82]. Recently, Pérez-Núñez et al. developed recombinant attenuated ASFV Arm/07/CBM/c2 deleted *CD2v* and *A238L* genes, which provided protection against the virulent Korean Paju strain [33].

Research groups in China have also published potential recombinant attenuated strains for the development of ASF vaccines. While certain vaccine candidates entered clinical trials, all testing was suspended due to safety concerns [83]. Among these strains, HLJ/18-7GD, which has six MGF genes and the *CD2v* (*EP402R*) gene deleted, was chosen from six other strains known to have attenuated and protective effects against the virulent ASF-HLJ/18 virus isolated in China in 2018. Importantly, this strain has demonstrated its safety even in pregnant sows [84]. More recently, a recombinant attenuated ASFV strain known as SY18ΔI226R was introduced. This strain features the deletion of the *I226R* gene from the SY18 strain. SY18ΔI226R exhibited both the absence of virulence and its effectiveness in protecting against the parental SY18 strain [85]. In another instance, the Chinese ASFV strain CN/GS/2018 was used to generate a highly attenuated seed (ASFV-ΔMGF110/360-9L) with two genes deleted: *MGF110-9L* and *MGF360-9L*. This recombinant strain successfully safeguarded all six pigs, ensuring their health and survival for 17 days post-challenge with the parental strain CN/GS/2018, whereas all six negative control pigs had perished by the 13th day post-challenge [86]. Furthermore, Qi et al. employed CN/GS/2018 as a backbone to create ASFV-GS-Δ18R/NL/UK, a three-gene-deletion mutant encompassing *DP148R*, *NL*, and *UK* genes. This recombinant strain exhibited strong attenuation and provided complete protection to immunized pigs against a homologous challenge [87].

**Table 1:** Potential candidates for developing ASF vaccines.

Type of Vaccine	Parental Strain	Genotype	Description	Challenge Strain	Safety	Efficacy	Reference
Natural live attenuated vaccine	NH/P68	I	ASFV NH/68 produced in porcine alveolar macrophage (PAM)	Homologous strain L60	Chronic ASF infections with side effects in the pigs	100%	[45,88]
				Heterologous strain Arm07		75%	[88]
	OUR T88/3	I		Heterologous strain Benin 97/1	-	85.7%	[46]
	OUR T88/3	I		Homologous strain OURT88/1	Fever (39.1–40 °C)	50–100%	[47,48]
	OUR T88/3	I		Heterologous strain DRC 085/10, Heterologous strain Uganda 1965	Fever (39.1–40 °C)	100%	[46,47]
	Lv17/WB/Rie1	II		Homologous strain HAD Latvian ASFV	Nonspecific clinical signs	100%	[49]
DNA and Virus-vectored vaccine	Spanish isolate E75	I	pCMV-UbsHAPQ, encoding the three abovementioned viral determinants (sHA, p54, and p30) fused to ubiquitin	Homologous strain Spanish E75	-	50%	[57]
	Ba71V	I	ASFV^Ublib^ contains 4029 clones representing 130 kbp of BA71V genome	Heterologous strain Spanish E75	No clinical signs and adverse effects	60%	[58]
	OUR T88/3 and Benin 1997/1	I	A pool of recombinant replication-deficient human adenovirus 5 (rAd) and recombinant modified vaccinia Ankara (MVA) containing codon-optimized ASFV genes, including *B602L*, *B646L*, *CP204L*, *E183L*, *E199L*, *EP153R*, and *F317L*, *MGF505-5R*, which are known to encode ASF antigens	Virulent strain OURT88/1	-	100%	[60]
Recombinant live attenuated vaccine	OUR T88/3	I	Deletion of the *DP71L* and *DP96R* genes, which affect ASFV virulence	Homologous strain OURT88/1	50% of the pigs had swollen joints	66%	[89]
	Georgia 2007/1	II	Deletion of the *B119L* (9 GL) gene, which affects ASFV virulence	Homologous strain Georgia 2007/1	The degree of virulence of the ASFV-G-Δ9GL virus depends on the amount of infectious virus. Safe at low doses	100%	[80]
	Georgia 2007/1	II	Deletion of *MGF505/360*(6) genes, which play a role in ASFV’s ability to evade host immunity	Homologous strain Georgia 2007/1	No clinical signs	100%	[69]
	Georgia 2007/1	II	Deletion of the *DP96R*(*UK*) and *B119L* (*9 GL*) genes, which affects ASFV virulence	Homologous strain Georgia 2007/1	No clinical signs	100%	[81]
	Benin 97/1	II	Deletion of the *MGF505/530/360* genes, which play a role in ASFV’s ability to evade host immunity	Homologous strain Benin 97/1	Transient fever (40–40.5 °C)	100%	[72]
	Benin 97/1	II	Deletion of the *DP148R* gene, which affects ASFV virulence	Homologous strain Benin 97/1	Mild clinical signs	83%	[68]
	BA71	II	Deletion of the *EP402R*(*CD2v*) gene, which affects ASFV virulence and hemadsorption	Homologous strain BA71; Heterologous strain E75 and Georgia 2007/1	No significant clinical signs	100%	[34]
	Benin 97/1	II	Deletion of the *MGF505/360* genes, which play a role in ASFV’s ability to evade host immunity	Homologous strain Benin 97/1	Fever (40.5–41.5 °C)	83%	[90]
	HLJ/18	II	Deletion of the *MGF505-1R/505-2R/505-3R/360-12L/360-13L/360-14L* genes, which play a role in ASFV’s ability to evade host immunity as well as *CD2v*, *9GL*, *DP148R*, and *UK* genes, which affect ASFV virulence	Homologous strain HLJ/18	No clinical signs; unlikely to revert to virulence	100%	[84]
	Georgia 2007/1	II	Deletion of the *I177L* gene, with unknown function	Homologous strain Georgia 2007	No clinical signs; unlikely to revert to virulence	100%	[73,76,77,78]
	Georgia 2010	II	Deletion of the *A137R* gene, with unknown function	Homologous strain Georgia 2010	Transient fever (≤103.4°F)	100%	[75]
	Georgia 2007/1	II	Deletion of the *I177l/LVR* genes, with unknown function	Homologous strain Georgia 2007	No clinical signs	100%	[35]
	Arm/07/CBM/c2	II	Deletion of the *CDv2/A238L* genes, which are involved in regulation of immune response	Heterologous virulent Korean Paju strain	No fever, no significant clinical signs	100%	[33]
	SY18	II	Deletion of the *I226R* gene, with unknown function	Homologous strain SY18	No clinical signs	100%	[85]
	CN/GS/2018	II	Deletion of the *MGF110-9L* and *MGF360-9L* genes, which affect ASFV virulence	Homologous strain CN/GS/2018	Fever (short duration and below 40.5 °C)	100%	[86]
	CN/GS/2018	II	Deletion of the *DP148R*, *DP71L*, and *DP96R* genes, which affect ASFV virulence	Homologous strain CN/GS/2018	No fever, low viral load in sera (<10^3^ HAD/mL)	100%	[87]

### 2.4. Future Strategies for ASF Vaccines

Recombinant attenuated ASFV strains are promising candidates for ASF vaccines; however, their safety and efficacy remain unclear. First, the ASF vaccine must ensure the safety of pigs of all ages and sexes. The impact of ASF on the immune system is still poorly understood, as it invades immune cells, including macrophages, and impairs the anti-viral action of the immune system. ASFV also contains proteins that inhibit immune factors or interfere with immune activity. In the trials, the vaccine was tested in fattening pigs, and only one strain was determined to be safe in pregnant sows. Some studies reported abortion in pregnant sows after inoculation with attenuated vaccine strains, and a high mortality in ensuing piglets. This can be explained by the ability of the virus to circulate for a long time in the body of pigs, and the possible impact of the virus on pigs with weak or immature immune systems (piglets). In addition, similar to naturally attenuated viruses, the virus could potentially persist in farms without control [91]. Another safety aspect is the risk of virus re-virulence when infecting the host; however, confirming this is time-consuming. In a recent study of the Arm/07 strain, the simultaneous existence of two strains belonging to two different genotypes, I and II, was predicted to have different toxicities. Arm/07/CBM/c4 and Arm/07/CBM/c2 possessed less than 98% identity, with 2800 variants in the whole genome. While the strain Arm/07/CBM/c2 was similar to strain Georgia 2007/1, the Arm/07/CBM/c4 strain was similar to other strains, such as OURT 88/3 [92]. Owing to similar hemadsorption and growth capacity in vitro, Arm/07/CBM/c2, but not Arm/07/CBM/c4, prevented IRF3 phosphorylation and IFN-β production in PAM cells. Whether these two strains are derived from an original strain or from a distinct infection, and whether the virulent and attenuated forms switch between each other, are important points that need consideration to determine the availability of vaccines. Quarantine policies have always been effective in preventing the spread of ASF. The long-term persistence of the virus in pigs, and its ability to spread from pork and porcine-derived products, has influenced strict regulatory policies in the global pork and porcine-derived products export business. The emergence of a disease-causing virus would disrupt the porcine food trade chain of the entire region. The threat of ASF becomes more complex and incredibly difficult to control when it is transmitted to Asia, which leads pork production worldwide [93]. Asian pork food systems typically show a higher number of small farms without improvements in farm management and sanitation behavior; additionally, food waste is salvaged as a nutrient source to decrease the cost, and fresh meat from wet markets is used, which raises risks of biosafety and hygiene control. Moreover, sick animals are illegally moved. These characteristics increase the risk of widespread infection. In the enduring endemic scenario, while grappling with the challenge of revitalizing the domestic livestock industry, mitigating losses for farmers, and meeting the high demand for pork, the Government of Vietnam made a significant shift in the official policy for the prevention and control of ASF. This shift involved allowing for partial culling on farms affected by ASF. Essentially, this change meant that pigs testing negative from these affected farms could be slaughtered or transported to other locations [94]. Therefore, the effective use of attenuated vaccines in these areas could contribute to limiting the enormous damage caused by ASFV. However, ASF prevention and control is relatively effective in Europe, Korea, and the United States, where strict quarantine policies and animal hygiene practices are established. Therefore, the risks and benefits of using attenuated vaccines in these countries and territories need to be weighed. Differentiating infected from vaccinated animals (DIVA), which encompasses the detection of both of antigens and antibodies, is also an issue that needs to be addressed with the ASF vaccines. A single-cycle vaccine can also improve the safety of attenuated vaccines. However, deleting genes involved in viral replication and assembly may affect the protective efficacy of the vaccine strain, and further studies are required on the functional genes of ASF. Removing these genes can also affect the ability of the virus to multiply in cells, making it difficult to produce vaccines later.

**Figure 2:**
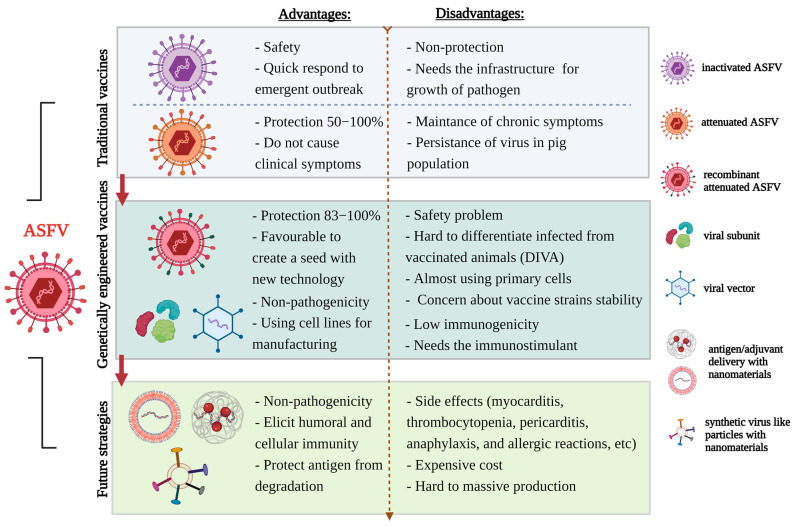
Overview of the strategies for ASF vaccine development. Conventional vaccines and currently used vaccines pose safety and efficacy problems. The application of nanomaterials may help improve the efficacy of non-pathogenic vaccines. This figure was created with BioRender.com.

Pathogen-free vaccines, such as nucleic acid-base and subunit vaccines, are potential solutions; however, advanced studies are necessary to improve protection. Nanotechnology-based vaccines could be potent for enhancing effectiveness (Figure 2). After the COVID-19 pandemic, the use of nanotechnology (lipid nanoparticles, viral vectors, and protein-assembled nanoparticles) in vaccine development has been adopted and commercialized with the aim of improving vaccine efficacy and subsequent viral control. The development of adjuvants and delivery platforms using nanoparticles that have unique chemical, physical, and biological properties provides us with flexible nano-sized constructs similar to natural antigens or pathogens [95,96]. These nanovaccine technologies can also be utilized in developing novel vaccines against ASFV and in improving the efficacy of existing swine vaccines [97,98,99]. Optimized nanoparticle designs (encapsulation or conjunction) have been employed for the persistent and stable release of antigens and the co-delivery of antigens and adjuvants, which supports the development of vaccine platforms with the capacity to stimulate robust innate and adaptive immune responses [100,101]. Nanotechnology platforms, such as liposomes, polymeric nanoparticles, and inorganic nanoparticles, have been utilized to enhance the stability of nucleic acid structures and the delivery of vaccines to target cells across cell membranes and nuclear membranes [102,103,104,105]. Generally, whole inactivated antigens and purified recombinant subunit proteins are poorly immunogenic. Nanovaccines can facilitate antigen processing and offer potent immunostimulatory platforms to regulate humoral and cellular immunity. Self-assembled synthetic virus-like particles (sVLPs) can be created with mosaically arrayed immunogenic antigens on the surface of nanoparticles; sVLPs could potentially increase virus protection and cross-protection, and are effective against viruses with complex genomes.

However, safety concerns remain the greatest hurdle in nanotechnology approaches to vaccine development. Severe adverse effects, including myocarditis, thrombocytopenia, pericarditis, anaphylaxis, allergic reactions, and inflammatory responses, may rarely occur with nano-based vaccines. With the approval of human nanovaccines, several nanosystems will undoubtedly be applied to combat ASFV. Therefore, securing and implementing a safety profile, such as via pharmacological, pharmacokinetic, and toxicological analyses, is necessary to its development and commercialization. Furthermore, nanovaccine commercialization studies should also focus on issues of stability, route of administration, and cost in terms of domestic pigs. Such nanotechnology-based vaccines against porcine infectious diseases, including ASF, could be potential candidates with enhanced efficacy.

## 3. Advanced Detection of ASFV

In this section, we discuss advanced diagnostic tools used for the development of ASFV for POC detection, including molecular and immunological tests to detect viral genomes, antigens, or antibodies, and focus on the factors that affect an accurate, sensitive, specific, user-friendly, equipment-free, fast, and multiplexed ASFV diagnostic system. We also describe how advanced technologies are integrated into the detection assays with signal-output and enhancers.

### 3.1. Hemadsorption Test

The HAD test is used as a basic diagnostic assay for ASFV isolation. Swine erythrocytes are allowed to adhere to the surface of peripheral blood mononuclear cells infected with ASFV. Blood samples or tissue suspensions from ASFV-suspected pigs are inoculated into primary leukocyte cultures isolated from the fresh defibrinated blood of naïve pigs. The assay is considered definitive for ASFV detection; however, it requires primary cell culture preparation, which takes more than 7 days to complete in order to discern the results. Therefore, it is only performed by reference laboratories. In addition, some ASFV strains do not display the HAD phenotype and test negative in the HAD assay. Deletion of the EP402R gene, which encodes the CD2v protein and expresses the protein responsible for HAD, has appeared in the lower-virulence natural ASFV mutants, which spread out in China, posing the possibility of false negative results. As a result of these limitations, molecular detection remains essential for the definitive detection of the virus in clinical samples.

### 3.2. Polymerase Chain Reaction

Molecular tests, such as PCR, real-time quantitative PCR (real-time PCR), and universal probe library-based PCR, are widely used to identify viral infections, with a potent sensitivity of 10–100 copies and high specificity. Real-time PCR-based tests are also predominantly employed worldwide to detect viral infections in suspected samples (nasopharyngeal swabs, sputum, whole blood, serum, and organ samples) in laboratories, and are considered the gold standard. PCR-based tests are highly sensitive and specific for ASFV identification and are useful under a wide range of circumstances, including unsuitability for virus isolation and antigen detection, such as putrefaction, degradation, or the inactivation of samples. Generally, methods of targeting conserved regions of the capsid protein p72 (*B646L gene*) have been developed, used, and validated in most PCR assays worldwide. The WOAH manual describes sensitive PCR methods as a general guideline and protocol, including conventional PCR and real-time PCR assays [106,107,108]. Real-time PCR assays for ASFV detection are based on the ASFV p72 (*B646L gene*) protein and whole genome sequences.

When performed using a nucleic acid extraction and PCR machine in the laboratory, the real-time PCR test has a turnaround time of approximately 4 h. However, depending on specimen collection, transportation, and processing, the entire process may take hours to days. Furthermore, these highly sensitive assays can lead to false positives due to cross-contamination and false negatives due to the presence of inhibitors or damaged nucleic acids. Therefore, novel PCR assays, which were developed by redesigning the primer sequence or forming a multiplex assay to improve efficacy and reduce processing time, showed higher sensitivity, specificity, and efficiency than those previously used globally [109,110,111,112,113,114]. Wang et al. added a new set of primers and probes, which cover the 5′ end region of p72, leading to potent diagnostic sensitivity and specificity; the limit of detection (LOD) was six copies of positive standard plasmids per reaction, or about 0.1–1 TCID_50_ of ASFV isolates per reaction, and ASFV was well distinguished from non-targeted common swine virus [109]. A more sensitive and economical real-time PCR assay was established using a lyophilized powder agent, including a pair of primers, a specific TaqMan probe, and Universal Master Mix II in a bottle [110]. This assay showed 10 times higher sensitivity (100 copies/µL) than that of the quantitative PCR assay recommended by WOAH (1000 copies/µL), and cost and time were saved by using lyophilized reaction mixture components. Trinh et al. developed a novel assay based on the conserved region of the ASFV *E183L* gene, which encodes the structural protein p54 [111]. This p54 real-time PCR had 2.63 copies/reaction of LOD and detected 15 different ASFV reference strains representing p72 genotypes I, II, and V. These novel PCR assays will be essential for the rapid detection and control of the ongoing global ASF epidemic.

Real-time PCR provides for the sensitive and specific molecular detection of pathogens and diseases; however, field utilization is limited because of sample shipping and an essential thermocycler, causing delays. Novel PCR assay applications, such as portable thermocyclers or on-site PCR cycling techniques for rapid detection, are being developed to make informed and timely decisions for early stage detection, treatment, and quarantine [115,116,117,118]. Daigle et al. described a highly sensitive and specific molecular assay using a portable thermocycler, Franklin^TM^ (Biomeme Inc., Philadelphia, PA, USA), for the rapid detection of ASFV [115]. Along with the on-site nucleic acid extraction kit, this system successfully evaluated nine clinical samples within 2 h, and the results were comparable to those obtained using laboratory methods [115]. Most recently, insulated isothermal PCR (iiPCR) has been used to detect universal ASFV or to differentiate genotypes I and II simultaneously [116,117,118]. iiPCR was carried out via the PCR reaction in one capillary vessel based on Rayleigh–Bénard convection, which is a naturally occurring fluid flow driven by buoyancy when a fluid layer is heated from the bottom end [119,120]. The convection of fluid materials in the vessel is cycled and completed through denaturation, annealing, and extension steps continuously and simultaneously by a gap in the temperature level. These technologies could serve as reasonable POC tools for the early detection and surveillance of ASFV.

### 3.3. Isothermal Amplification-Based Molecular Diagnostics

Real-time PCR is widely recognized as the gold standard for detecting and confirming the presence of ASFV genomic DNA in samples, attributed to its exceptional sensitivity and accuracy. Nevertheless, executing real-time PCR in resource-constrained environments can pose challenges because it requires the use of thermal cycler equipment to amplify specific DNA fragments through denaturation, annealing, and extension. Nonetheless, conducting on-site testing in such constrained settings is of paramount importance for early stage quarantine and the monitoring of highly contagious diseases such as ASFV. The isothermal nucleic acid amplification method is a promising molecular diagnostic tool that swiftly induces strand displacement and extension without the need for thermal cycling. This technique proves ideal for portable instruments and extensive field testing because of its brief amplification period and low power consumption, all of which deliver remarkably sensitive outcomes [121,122]. Furthermore, diverse primer designs and processing techniques involving enzyme-assisted or free amplification can be employed to target genes of various sizes and lengths. This flexibility enables in-field detection of specific nucleic acids through isothermal amplification, ensuring the attainment of results with heightened sensitivity.

Recently, various novel isothermal amplification-based molecular diagnostics have been developed, including loop-mediated isothermal amplification (LAMP), rolling circle amplification (RCA), recombinase polymerase amplification (RPA), hybridization chain reaction, and cross-priming amplification [123,124,125]. These methods exploit a separate enzyme or the strand-displacement activity of specific DNA polymerases to generate ssDNA and amplify a target sequence in an exponential, linear, or cascading manner for less than an hour after primer binding. To quantitatively analyze the amplicons produced using isothermal amplification methods, several techniques have been employed, including intercalating dyes, fluorescence-labeled probes, measuring by-products (e.g., magnesium pyrophosphate), and absorbance changes of the amplicon products.

To identify ASFV, LAMP-based testing is potentially the most promising method because of its speed, sensitivity, simplicity, ease of operation, and cost-effectiveness [126]. The LAMP test utilizes a strand-displacement DNA polymerase and generates a stem-loop DNA structure. The resulting LAMP amplicons can be identified using agarose gel electrophoresis, fluorometry, or even through naked-eye observation of color changes. Detection can be achieved through real-time or end-point methods [127,128,129]. James et al. successfully demonstrated the viability of developing a reliable LAMP assay for the rapid field detection of ASFV, all without the need for thermal cycling [130]. This LAMP assay, which targeted topoisomerase II, exhibited an LOD of at least 330 genome copies, effectively identifying representative ASFV isolates without cross-reacting with two classical swine fever isolates. Furthermore, the process of DNA amplification via LAMP can be tracked through a diverse array of colorimetric indicators, streamlining its application for POC testing. To precisely distinguish outcomes from color changes and confirm on-site detection, Wang et al. devised a real-time LAMP and visual colorimetric assay using a chromogenic pH-responsive dye. This innovation enabled accurate result interpretation. The assay, designed to target the *A78R* gene of ASFV, attained an LOD of 30 copies per μL, surpassing the performance of the LAMP assay developed by James et al. [130,131]. Furthermore, studies that directly employed LAMP reactions on crude samples such as whole blood or serum, without the need for additional gene extraction, have showcased considerable potential for application in resource-limited POC settings [132,133]. Tran et al. conducted a colorimetric LAMP assay using crude samples from infected domestic pigs in Vietnam without a prior DNA extraction step, and showed 100% sensitivity and specificity within 30 min at 60 °C [133]. This direct colorimetric LAMP assay was as sensitive as the recommended real-time PCR, despite using clinical serum samples without DNA extraction.

Recombinase-based isothermal reactions (recombinase polymerase amplification (RPA) and recombinase-aided amplification (RAA)) are conducted at low temperatures (37–42 °C), and thermal cyclers are not used. The amplicon signals could be detected in 10–20 min using gel electrophoresis, real-time or end-point monitoring, or lateral flow detection (LFD) assays. RPA and RAA, developed by TwistDx (Cambridge, UK) and Qitian (Wuxi, China), respectively, have been commercialized and applied to detect pathogens and viruses [134,135]. In comparison to WOAH real-time PCR for ASFV detection, the sensitivity values of RPA and RAA were 96.59% and 97.73%, respectively, with both showing excellent specificity at 100% [136]. In the field diagnosis of pathogens, an LFD is commonly portable and easy to use. The RPA/RAA isothermal amplification and lateral flow reaction were completed within 1 h at low temperatures, which was faster than other methods that required several hours to days and sophisticated equipment [137,138]. Wen et al. developed a quantum dot microsphere-based fluorescence strip assay for ASFV genome detection using RAA in 25 min with a detection limit of one copy for ASFV plasmid templates and 100 copies/g for pork samples per reaction [139].

These studies shed light on the fact that the novel isothermal amplification-based assay holds the potential to supplant real-time PCR for the on-site amplification of ASFV DNA, eliminating the need for thermal cycling. Furthermore, this assay can be integrated with optical techniques such as fluorescence, luminescence, absorbance, or antigen-antibody LFD methods. Such integration facilitates the efficient monitoring of amplicons and result confirmation, culminating in rapid and reliable ASFV detection with remarkable sensitivity and high specificity. These assays are particularly well-suited for POC testing due to their user-friendly nature, enabling the generation of results without the need for intricate scientific interpretation.

### 3.4. CRISPR

Clustered, regularly interspaced short palindromic repeats (CRISPR)-associated proteins (CRISPR-Cas), engineered via a gene-editing technique, hold promise for next-generation pathogen or virus diagnostic technologies [140,141]. The application of these tools in molecular diagnostics, including Cas9, Cas12a, Cas13a, and Cas14a systems, has been broadly developed for genetic ASFV detection platforms [142,143]. The CRISPR–Cas-based diagnostic system combines the ability of target sequence-specific recognition and cleavage with nucleic acid amplification, including PCR and isothermal amplification, to enhance accuracy and sensitivity [144,145]. Furthermore, to facilitate the direct on-site diagnosis of ASF, this CRISPR/Cas recognition technique has also been integrated into an LFD platform or portable readers to introduce a POC system [146,147,148,149]. Wang et al. combined CRISPR/Cas9 into a lateral flow assay (CASLFA) and utilized it to detect the *B646L* gene sequence from the ASFV [150]. Using serum samples from ASFV-suspected swine (*n* = 110), the CASLFA method identified 27 ASFV-infected swine samples with identical results to the real-time PCR method within 40 min, including DNA extraction and detection [150]. Cas12a, an RNA-guided DNA endonuclease, induces non-canonical trans-cleavage activity upon recognizing dsDNA. By combining the trans-cleavage activity of Cas12a, it has been developed for a variety of molecular diagnostic technologies. Tian et al. designed two heterologous CRISPR-Cas12a/Cas13a systems in one pot to exploit orthogonal cleavage activity for the dual-gene detection of SARS-CoV-2 (O and N genes) and ASFV (*B646L* and housekeeping *ACTB* gene) [151]. By introducing pre-amplification using RPA methods and a handheld smartphone-based fluorescence device, a rapid, accurate, and sensitive POC detection platform was achieved. Ki et al. introduced a novel diagnostic approach based on colorimetric absorbance shift. This method combined the CRISPR-Cas12a target recognition system with an enzyme urease-based reaction to establish a highly sensitive POC system for detecting ASF [152]. Upon the trans-cleavage activity of the CRISPR-Cas12a complex in the presence of ASF-specific DNA, the released urease induced a cascade reaction through urea hydrolysis. This reaction led to shifts in solution pH and color, allowing for a colorimetric readout.

By integrating advanced nucleic acid extraction and amplification techniques, such as isothermal amplification methods, with CRISPR diagnostic systems, the assay’s performance is elevated, and its range of application expands, making it a potent tool for efficient POC testing. Moreover, nano-devices and probes contribute to solutions for colorimetric confirmation strategies, enhancing the portability and universality of diagnostic approaches. Despite their relatively short development timeline, CRISPR-based diagnostics are rapidly advancing. CRISPR technology brings unprecedented advantages for creating innovative qualitative and quantitative molecular diagnostic methods, presenting a promising alternative to conventional assays.

### 3.5. Antibody-Based Immunoassay

Antibody-based immunoassays have long been essential for disease detection and monitoring. This is the most commonly used method as it is relatively simple and inexpensive. Generally, ASFV infection elicits a strong humoral immune response, and the retention of ASFV-specific antibodies indicates a current or previous infection. Several recommendations have been made by the WOAH for ASFV antibody diagnostics prior to transport and for disease surveillance, including in pigs and wild boars. However, in general, serological diagnoses could yield false negatives; low antibody levels result in low sensitivity, especially in pigs that died in the early stage of infection (within 7 days of infection) or from acutely virulent strains. To develop a powerful diagnostic method in the field, fluorescence-based and nano–microparticle techniques have been proposed to enhance sensitivity and POC testing.

ELISA is the most used laboratory method for screening and detecting the prevalence of infections. It is available for high-throughput screening and provides results within 2–5 h. Soluble antigens isolated from cells infected with the adapted virus can be used for indirect ELISA, but a biosafety level (BSL)-3 facility is required, making it unsuitable for in-field or lower BSL labs. Recombinant ASFV proteins, including p72, p30, and p54, can be used as viral antigens to replace native antigens and secure comparable or improved sensitivity and specificity in ELISA for ASFV antibody diagnosis [153,154,155,156,157]. Mutants that cause nonlethal, subacute, chronic, or persistent infections may also exhibit a non-HAD phenotype, resulting in significant economic losses on farms. The CD2v protein, an outer-membrane glycoprotein and hemagglutinin, is required to mediate HAD [158,159,160]. Thus, Lv et al. developed a dual ELISA for distinguishing CD2v-unexpressed low-virulence ASF mutants using CD2v and p30 recombinant proteins [161]. The dual ELISA kit differentiated wild-type ASFV-positive and -negative serum samples in the same way as the ASFV VP72 antibody-blocking ELISA kit from Ingenasa (INgezim 11. PPA.K3, Madrid, Spain), and CD2v-unexpressed ASFV infection. In addition, microplate-based immunoassays, such as semi-automated or one-step sandwich-type assays, have been developed to expedite the time-consuming and sensitive detection process of ASFV in serum [157,162,163,164].

Commonly used serological ASFV antibody diagnostics in the laboratory include indirect immunoperoxidase tests, indirect fluorescent antibody tests, and immunoblotting. These tools are time-consuming and require specific equipment and technical skills, making them challenging to use as a POC system. Lateral flow analysis typically uses gold nanoparticles as a naked-eye visualizer and is the simplest, fastest, most portable, and most cost-effective method that is easy to use and interpret, making it suitable for POC testing [165,166]. Wan et al. developed a dual LFA with p30 and p72 proteins, which had good antigenicity and were expressed in the early and late stages of ASFV infection, respectively [167,168]. The color development of the detection lines of p30 and p72 indicated the infection status of the host simultaneously, monitoring the antibody levels in the sera. In addition, to improve sensitivity, LFAs that replace gold nanoparticles with quantum dots with strong luminescence, high stability, and quantum yield have been developed [169].

ASF-specific antigen and antibody diagnostic assays play a critical role in the monitoring and surveillance of infections. Individuals who have survived ASFV infection, especially in its chronic form, can exhibit positive antibody responses. Even after 30–40 days post-infection, antibodies might persist in these individuals, despite PCR results typically being negative. These circumstances can facilitate the continued transmission of the virus and sustain its presence on farms. Hence, when introducing or relocating swine, it becomes imperative to ascertain that the farm has a demonstrable negative antibody status. Employing antibody-based immunoassays aids in confirming the presence of infection and comprehending the path of transmission.

### 3.6. Future Direction of ASFV Diagnostics

ASFV is highly contagious and infectious, with a mortality rate of up to 100% in swine over a short period [170,171]. Given the rapid onset and high mortality rate, antibody-based detection tests may be impractical in the field. Therefore, the rapid and accurate diagnosis of viral antigens in on-site specimens is essential for efficient POC testing (Table 2). Nanotechnology and DNA technologies, including nanoscale materials and processes, have received significant attention for their use in developing infectious disease diagnostic platforms that offer improved sensitivity and accuracy, shorter turnaround times, and early detection [172]. These diagnostic technologies have transitioned from the laboratory environment and proof-of-concept stages to the actual approval process using clinical samples. However, commercializing these systems faces challenges, including issues related to the homogeneous mass production of nano-sized products, reproducible results, user-friendliness, and cost-effectiveness. Moreover, improved sensitivity must be accompanied by the development of a simple, well-defined portable device for signal readouts, employing fluorescence or absorbance.

In addition, there has been recent progress in the development of ASF vaccines using recombinant attenuated vaccines [77]. However, safety concerns remain a significant consideration. Notably, the exploration of live-attenuated vaccines is ongoing. Vaccination is becoming increasingly acceptable as an alternative to surveillances through the eradication of infected and exposed species in countries where the infection is widespread. Therefore, antibody-based diagnostics are essential to evaluate the immune responses after vaccination, and developing diagnostic kits for DIVA will also become a focus. DIVA strategies can be established as either molecular or immunoassay diagnostics: multiplex real-time PCR targeting the *B646L* gene of wild-type ASFV and the deleted gene of the vaccine, or deleted gene-encoded protein-specific antibody diagnostics using ELISA or LFA. These DIVA diagnostics can not only assess the performance of the vaccine, but also simultaneously monitor infection during vaccination. For ASFV, which requires strict biosecurity control, DIVA allows vaccination while retaining the possibility of serological surveillance for the infection.

## 4. Conclusions

ASF remains a challenge worldwide, and traditional vaccines do not demonstrate sufficient protective efficacy. The commercially available vaccine is a recombinant attenuated vaccine developed from the ASFV-G-ΔI177L strain. In addition, some recombinant attenuated ASFV strains may hold promise for future vaccine production. Currently, recombinant attenuated vaccines have shown effective protection, but safety issues need to be addressed in further studies. Meanwhile, genetically engineered vaccines, such as DNA and subunit vaccines, have not yet performed well but offer significant advantages in the production and utilization process. The application of nanotechnology in designing novel vaccines with secure safety profiles could be a potential direction against ASFV. Research on diagnostic technology, alongside the development of novel vaccines, is essential to rapidly control and prevent ASFV infection. The WOAH manual recommends identifying methods for ASFV antigen and antibody testing in laboratory facilities with skilled technicians; however, POC diagnostics are required for effective quarantine and on-site prevention. To achieve an LOD comparable to PCR without the need for a sophisticated facility, various isothermal amplification techniques have been proposed, and sensitivity and specificity have been improved using CRISPR. Additionally, facilitating optical signal readout (fluorescence, colorimetry, gold nanoparticles, quantum dots, etc.) has been proposed with portable devices or platforms. DIVA diagnostics can also be used to monitor and focus serological surveillance, leading to a reduction in indiscriminate slaughter and costs. Development and innovation in prophylactic vaccines and diagnostic systems will contribute to effective control in regions where ASFV is endemic, reducing the possibility of ASFV occurring in ASF-free regions.

## Figures and Tables

**Table 2:** Point-of-care diagnostic comparison for the detection of the ASFV.

Technique	Device	Target Analyte	LOD	Time	Ref
PCR	Real-time PCR with portable thermocycler	P54 (*E183L*)	Comparable to laboratory real-time PCR	2 h	[115]
	Portable iiPCR	P72 (*B646L*)	Comparable to real-time PCR	1.5 h	[116]
	iiPCR, POCKIT^TM^ Micro Duo	AP endonuclease (*E296R*)	20 copies/μL	40 min	[117]
Isothermal amplification	LAMP-colorimetric	P10 (*A78R*)	30 copies/μL	1 h	[131]
	LAMP-colorimetric	P72 (*B646L*)	10 copies/reaction	45 min	[132]
	LAMP-colorimetric	Topoisomerase II gene (*P1192R*)	1 HAD_50_/mL in viral genomic DNA 10 HAD_50_/ mL in serum samples	30 min	[133]
	RPA	*B646L* gene	3.5 copies/μL	7 min	[134]
	Real-time RPA	P30 (*CP204L*)	5 × 10^1^ copies/reaction	6 min	[135]
	RPA and RAA	P72 (*B646L*)	93.4 and 53.6 copies/reaction	16 min	[136]
	RPA-LFA	P72 (*B646L*)	150 copies/reaction	10 min	[137]
	RPA-LFD	*K205R* gene	1 × 10^2^ copies/reaction	30 min	[138]
	RAA-QD-LFA	P72 (*B646L*)	100 copies/g for DNA extracts	25 min	[139]
CRISPR	CRISPR/Cas12a-LAMP-Fluorescent	P72 (*B646L*)	1 copies/μL	50 min	[142]
	CRISPR/Cas12a-RPA-Fluorescent	P72 (*B646L*)	2 copies/reaction	30–40 min	[143]
	CRISPR/Cas12a-LAMP-Fluorescent	P72 (*B646L*)	2 copies/μL reaction	1 h	[144]
	CRISPR/Cas12a-RPA-LFA	PP220 polyprotein (*CP2475L*)	200 copies of viral genome	90–100 min	[146]
	CRISPR/Cas12a- Fluorescenct	P72 (*B646L*)	100 fM (5.7 × 10^7^ copies/mL)	2 h	[147]
	CRISPR/Cas12a-LFA	P72 (*B646L*)	20 copies/reaction	1 h	[148]
LFA	PenCheck^®^ LFA kit	P30 (*CP204L*)	107.80 TCID_50_/mL	25 min	[165]
	Colloidal gold-LFA	P30 (*CP204L*)	2.16 ng of P30	5–7 min	[166]
	Colloidal-gold dual immunochromatography strip	P30 (*CP204L*) and P72 (*B646L*) Ab	Equivalent to commercial ELISA kits (1:256 dilution to positive sample)	5–10 min	[167]
	QDs-based fluorescent LFA	CD2v (*EP402R*) Ab	1:5.12 × 10^5^ dilution to positive serum	20 min	[169]

Abbreviations: LOD, limit of detection; PCR, polymerase chain reaction; Ref, reference; iiPCR, insulated isothermal PCR; LAMP, loop-mediated amplification; HAD, hemadsorption; RPA; recombinase polymerase amplification; RAA, recombinase-aided amplification; LFA, lateral flow assay; QD, quantum-dot; CRISPR, clustered regularly interspaced short palindromic repeats.
